# The FinR-regulated essential gene *fprA*, encoding ferredoxin NADP^+^ reductase: Roles in superoxide-mediated stress protection and virulence of *Pseudomonas aeruginosa*

**DOI:** 10.1371/journal.pone.0172071

**Published:** 2017-02-10

**Authors:** Siriwan Boonma, Adisak Romsang, Jintana Duang-nkern, Sopapan Atichartpongkul, Wachareeporn Trinachartvanit, Paiboon Vattanaviboon, Skorn Mongkolsuk

**Affiliations:** 1 Department of Biotechnology, Faculty of Science, Mahidol University, Bangkok, Thailand; 2 Laboratory of Biotechnology, Chulabhorn Research Institute, Bangkok, Thailand; 3 Department of Biology, Faculty of Science, Mahidol University, Bangkok, Thailand; 4 Center of Excellence on Environmental Health and Toxicology, CHE, Ministry Of Education, Bangkok, Thailand; 5 Program in Applied Biological Sciences: Environmental Health, Chulabhorn Graduate Institute, Bangkok, Thailand; 6 Center for Emerging Bacterial Infections, Faculty of Science, Mahidol University, Bangkok, Thailand; University of North Dakota, UNITED STATES

## Abstract

*Pseudomonas aeruginosa* has two genes encoding ferredoxin NADP(+) reductases, denoted *fprA* and *fprB*. We show here that *P*. *aeruginosa fprA* is an essential gene. However, the Δ*fprA* mutant could only be successfully constructed in PAO1 strains containing an extra copy of *fprA* on a mini-Tn7 vector integrated into the chromosome or carrying it on a temperature-sensitive plasmid. The strain containing an extra copy of the ferredoxin gene (*fdx1*) could suppress the essentiality of FprA. Other ferredoxin genes could not suppress the requirement for FprA, suggesting that Fdx1 mediates the essentiality of FprA. The expression of *fprA* was highly induced in response to treatments with a superoxide generator, paraquat, or sodium hypochlorite (NaOCl). The induction of *fprA* by these treatments depended on FinR, a LysR-family transcription regulator. In vivo and in vitro analysis suggested that oxidized FinR acted as a transcriptional activator of *fprA* expression by binding to its regulatory box, located 20 bases upstream of the *fprA* -35 promoter motif. This location of the FinR box also placed it between the -35 and -10 motifs of the *finR* promoter, where the reduced regulator functions as a repressor. Under uninduced conditions, binding of FinR repressed its own transcription but had no effect on *fprA* expression. Exposure to paraquat or NaOCl converted FinR to a transcriptional activator, leading to the expression of both *fprA* and *finR*. The Δ*finR* mutant showed an increased paraquat sensitivity phenotype and attenuated virulence in the *Drosophila melanogaster* host model. These phenotypes could be complemented by high expression of *fprA*, indicating that the observed phenotypes of the Δ*finR* mutant arose from the inability to up-regulate *fprA* expression. In addition, increased expression of *fprB* was unable to rescue essentiality of *fprA* or the superoxide-sensitive phenotype of the Δ*finR* mutant, suggesting distinct mechanisms of the FprA and FprB enzymes.

## Introduction

*Pseudomonas aeruginosa* is one of the most common opportunistic bacterial pathogens that cause deadly infections in patients with impaired immune systems or in critical condition. Nosocomial infections caused by *P*. *aeruginosa* are increasing worldwide [1, 2]. The ability of a pathogen to overwhelmingly invade the host is often associated with its ability to rapidly adapt and evade or overcome host immune systems. During pathogen-host interactions, several transcriptional regulators are differentially expressed to fine-tune gene expression networks required for adaptive responses to host-generated stresses [3]. Reactive oxygen species (ROS) are key components of host innate immune responses generated within the phagolysosomes of phagocytes to attack invading microbes. Additionally, normal aerobic metabolism produces ROS as by-products [4]. Consequently, bacteria have evolved mechanisms to protect themselves from oxidative stress. An array of either antioxidant enzymes, such as catalases, superoxide dismutases, and thiol peroxidases or antioxidant molecules, such as glutathione and vitamins are involved in removal of ROS. In addition, there are extensive repaired and rebuilding systems for oxidatively damaged molecules, such as iron-sulfur cluster biosynthesis (Isc), DNA repair (the Mut systems) and proteins repair (methionine sulfoxide reductases, Msr). These systems are necessary for bacterial survivals under stressful conditions [5, 6]. Various transcriptional regulators are involved in coordinating the complex processes of sensing and responding to stresses. The LysR-type transcriptional regulators (LTTRs) represents the most abundant type of transcriptional regulator with an N-terminal DNA-binding helix-turn-helix motif and a C-terminal co-inducer-binding domain as conserved structures. LTTRs exhibit a negative autoregulation and regulate a diverse set of genes, including those involved in virulence, metabolism, quorum sensing and motility [7–15]. A major regulator in hydrogen peroxide (H_2_O_2_) defense is OxyR, a LysR-type transcriptional regulator, while the transcription factor SoxR triggers a global stress response against superoxide anions as well as redox cycling drugs [16–19]. Many proteobacterial genomes contain another LysR-type oxidative stress sensing transcriptional regulator, FinR, which is located next to *fprA* (ferredoxin NADP^+^ reductase, Fpr), an enzyme catalyzing the reversible electron-transferring reaction between NADPH and one-electron carriers such as ferredoxin or flavodoxin. The enzymes are important in maintain NADP(+)/NADPH ratio. Fpr also catalyzes the irreversible electron transfer in diaphorase reaction which drives the oxidation of NADPH in a wide variety of adventitious electron acceptors [20]. In bactria, Fpr has been shown to control the ratio of NADP(+)/NADPH. Fpr participates in many cellular processes, including iron acquisition, iron-sulfur cluster biogenesis and oxidative stress defense [21, 22]. FinR is required for the induction of *fprA* expression upon exposure to superoxide anion stress generated by paraquat [21, 23–25]. However, *Escherichia coli fpr* is a member of the SoxRS regulon, and inactivation of *fpr* increases sensitivity to paraquat [26, 27].

*Pseudomonas putida* KT2440 contains at least two genes encoding Fpr, namely *fprA* and *fprB* [23, 28]. The *fprA* mutant confers high sensitivity to oxidative and osmotic stresses, while the *fprB* mutant is susceptible only to high osmotic conditions [23, 28]. Like *P*. *putida*, *P*. *aeruginosa* PAO1 possesses both *fprA* and *fprB*. Two types of Fprs have their preferred electron transport and redox partners. FprA achieves higher catalytic efficiency when flavodoxin is its redox partner. FprB is important in defenses against multiple stresses including metal, oxidative, and osmotic stresses and is required for the full function of iron-sulfur cluster ([Fe-S])-containing enzymes via its redox partner, Fdx2, involving in the ISC [Fe-S] biogenesis system [29]. For example, in an iron storage complex, the [Fe-S] cluster of bacterioferritin-associated ferredoxin (Bdf) transfers electrons to the heme in bacterioferritin (BfrB) and promotes the release of Fe^2+^ from BfrB by mediating electrons from FprA to BfrB [30]. Moreover, roles for FprA in sulfate assimilation and siderophore biosynthesis in pseudomonads have been characterized [31]. The expression of *fprB* could be induced by exposure to oxidative stress in an [Fe-S] biogenesis regulator IscR-dependent manner [29]. The physiological function of *P*. *aeruginosa* FprA remains mysterious due to unsuccessful construction of the *fprA* mutant [31, 32]. This observation raised the possibility that the activity of FprA is essential for bacterial viability. In this communication, *P*. *aeruginosa fprA* was shown to be essential and was determined to be regulated by FinR.

## Results and discussion

### *fprA* is an essential gene in *P*. *aeruginosa*

*P*. *aeruginosa* PAO1 has two annotated *fpr* genes, *fprA* (PA3397) and *fprB* [33]. An open reading frame located next to *fprA* in the opposite orientation was annotated as a putative LysR-type transcriptional regulator, FinR (PA3398). *P*. *aeruginosa* FinR shares 80.8% and 80.5% amino acid sequence identity with FinR from *P*. *putida* and *Azotobacter vinelandii*, respectively (S1 Fig). Several attempts to construct the *fprA* mutant in pseudomonads have been met with mixed results. No mutants were obtained in *P*. *aeruginosa*, but a mutant was constructed in *P*. *putida* [31, 32]. These observations suggest the essentiality of *fprA* in PAO1. We made several unsuccessful attempts to construct either insertion inactivation or deletion *fprA* mutants. Hence, the notion of the essentiality of *fprA* was tested. A new PAO1 parental strain was constructed that had an extra copy of *fprA* cloned into a mini-Tn7 vector [34], and the recombinant Tn7T*-fprA* transposed into the PAO1 chromosome *att*Tn7 site, giving PAO1::Tn7T-*fprA*. The antibiotic marker of the mini-Tn7 vector was removed by the Flp-FRT system [35]. *fprA* gene deletion by allelic exchange was made by electroporating pUCΔ*fprA*::Gm^r^ (Table 1) into PAO1::Tn7T-*fprA* and selecting for gentamicin resistance (Gm^r^). Several Gm^r^ and carbenicillin susceptible (Cb^s^) colonies were screened by PCR and found to have deleted the functional copy of the chromosomal *fprA* gene. The Δ*fprA* mutant (Δ*fprA*::Tn7T-*fprA*) was successfully constructed. In the control strain, which contains only the mini-Tn7 vector (PAO1::Tn7T), no Gm^r^ transformants or *fprA* mutants were recovered (Table 1). The results support the notion that *fprA* is an essential gene in PAO1.

**Table 1:** Efficiency of *fprA* deletion in *P*. *aeruginosa* strains carrying an extra copy of various genes.

*P*. *aeruginosa* strains	Efficiency of *fprA* deletion^a^
PAO1::Tn7T	ND
PAO1::Tn7T-*finR*	ND
PAO1::Tn7T-*fprA*	1.6 × 10^2^
PAO1::Tn7T-*fprB*	ND
PAO1::Tn7T-*fdx1*	2.1 × 10^1^
PAO1::Tn7T-*fdx2*	ND
PAO1::Tn7T-*rnfB*	ND
PAO1::Tn7T-*fdxA*	ND

^a^ Indicated strains of PAO1 were transformed with 1 μg pΔ*fprA*::Gm^r^ plasmid using electroporation. The transformants with *fprA* deletion were selected by the Gm^r^ and Cb^s^ phenotypes. The efficiency of *fprA* deletion is defined as the number of Δ*fprA* mutant obtained per 1 μg pUCΔ*fprA*::Gm^r^ plasmid. The data shown are means from triple independent experiments. ND, not detectable.

**Fig 1:**
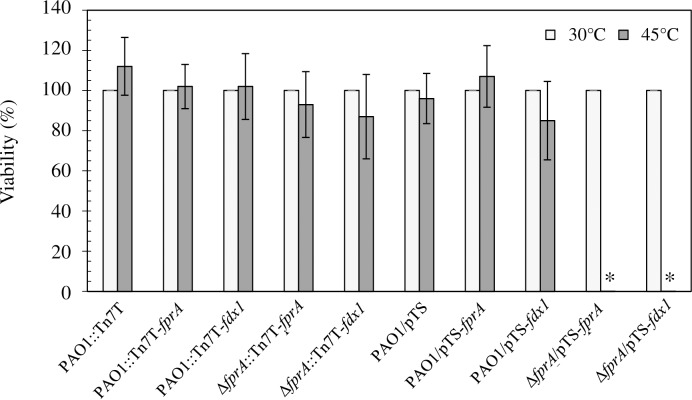
*fprA* is an essential gene in *P*. *aeruginosa*. The viability of exponential-phase cultures of *P*. *aeruginosa* PAO1 and Δ*fprA* mutant strains harboring an extra copy of *fprA* or *fdx1* was determined using viable cell count on LB agar plates incubated at either 30°C or 45°C. The viability is expressed as a percentage of the CFU of the tested strain over the CFU of the PAO1::Tn7T or PAO1/pTS control.

An independent approach was conducted to assure the essentiality of *fprA* in *P*. *aeruginosa* using a plasmid vector with a temperature-sensitive origin of replication that has been recently developed in *P*. *aeruginosa* [36]. We firstly constructed a temperature-sensitive replication plasmid by cloning the temperature-sensitive replicon mSF^*ts1*^ from pSS255 [36] into the broad-host-range vector pBBR1MCS-4 [37], yielding pTS. The plasmid can be maintained at 30°C but not at the non-permissive temperature of 45°C. The full-length *fprA* was cloned into the plasmid pTS, generating pTS-*fprA*. Transformants harboring pTS-*fprA* were grown and maintained at 30°C. Growing bacterial cultures at the non-permissive temperature of 45°C resulted in the loss of pTS-*fprA*. pUCΔ*fprA*::Gm^r^ was introduced into PAO1 harboring pTS-*fprA*, and Gm^r^ transformants were selected and screened for double crossing over and marker exchange events, giving Δ*fprA*::Gm^r^/pTS-*fprA*. The Δ*fprA*::Gm^r^/pTS-*fprA* mutant strain had normal growth at 30°C. This mutant strain could not grow on either an agar plate or in LB broth medium when the incubation temperature was shifted to the non-permissive temperature of 45°C for pTS-*fprA*, indicating the essentiality of *fprA* (Fig 1). The results confirmed that *fprA* is an essential gene that is required for the growth of PAO1. Although *P*. *aeruginosa* FprA shares the greatest amino acid sequence identity with FrpA from *P*. *putida* and *A*. *vinelandii* (S1 Fig), there is no evidence suggesting that it is essential in these two bacteria [31, 38].

Since PAO1 has both *fprA* and *fprB*, we tested the potential cross-functional complementation between *fprB* and *fprA*. Similar approaches that were successfully used to construct the Δ*fprA* mutant were applied to test cross-complementation between *fprA* and *fprB*. A PAO1::Tn7T-*fprB* strain carrying an extra copy of *fpr*B was used for Δ*fprA* mutant construction using pUCΔ*fprA*::Gm^r^. After several attempts, no *fprA* mutant was obtained. This indicated the essential function of *fprA* for bacterial growth and showed that expression of *fprB* could not complement *fprA*. This suggests that FprA and FprB have different biochemical and physiological functions. Fpr have essential functions in maintenance of the NAD(P)/NAD(P)H ratio via their reactions with ferredoxins and flavodoxins. In the *fprA* mutant, alterations in the ratio of reduced/ oxidized ferredoxins could contribute to the mutant lethality under tested conditions.

### The requirement for *fprA* could be complemented by *fdx1* expression

Fpr catalyzes reversible electron transfer between NADPH and electron carriers such as ferredoxins (Fdx), thereby maintaining a balance between NADPH and reduced Fdx pools. Since Fpr is important in maintaining reduced Fdx, we determined whether the expression of *fdx* genes could suppress the essentiality of *fprA*. PAO1 has several genes encoding Fdx of different families, e.g., *fdx1* (PA0362), encoding two[4Fe-4S]-containing bacterial ferredoxin; *fdx2* (PA3809) (a member of the *isc* operon that is involved in iron-sulfur cluster biogenesis), encoding a [2Fe-2S]-containing ferredoxin; *rnfB* (PA3490), encoding a ferredoxin-like protein; and *fdxA* (PA3621), encoding a [4Fe-4S] cluster-containing ferredoxin [33],[6, 39–42]. We tested whether the essentiality of the *fprA* gene was due to its Fdx1 redox partners. Using a similar strategy as used for the construction of the *fprA* mutant, *P*. *aeruginosa* PAO1 strains were constructed with an extra copy of *fdx1* (PAO1::Tn7-*fdx1*), *fdx2* (PAO1::Tn7-*fdx2*), *rnfB* (PAO1::Tn7-*rnfB*) or *fdxA* (PAO1::Tn7-*fdxA*) and used to test whether Δ*fprA* mutants could be constructed with a suicide plasmid pUCΔ*fprA*::Gm^r^. The *fprA* mutant construction was accomplished only in the parental strains PAO1::Tn7-*fdx1* and PAO1::Tn7T*-fprA* (Table 1). In other parental strains tested, no *fprA* mutant could be recovered. The functional complementation of *fprA* by *fdx1* was confirmed by the fact that the Δ*fprA* mutant harboring pTS-*fdx1* could grow at 30°C and at the non-permissive 45°C (Fig 1). This finding indicated that expression of *fdx1* can suppress the essential function of *fprA* and permit the growth of the Δ*fprA* mutants. It is likely that Fdx1 functions as a redox partner of FprA. We speculate that deletion of *fprA* severely affects the redox status of Fdx1 by shifting the ratio between reduced and oxidized forms. Increased expression of *fdx1*, either from Tn7T-*fdx1* or pTS-*fdx1* in the mutant was sufficient to compensate for the loss of FprA function by restoring the ratio of reduced/oxidized ferredoxins to a viable levels for *P*. *aeruginosa*. Fdx1 has been shown to be essential for the viability of PAO1 [40]. The physiological role of Fdx1 in *P*. *aeruginosa* remains unclear.

### *fprA* and *finR* expression increased in response to exposure to paraquat and NaOCl

**Fig 2:**
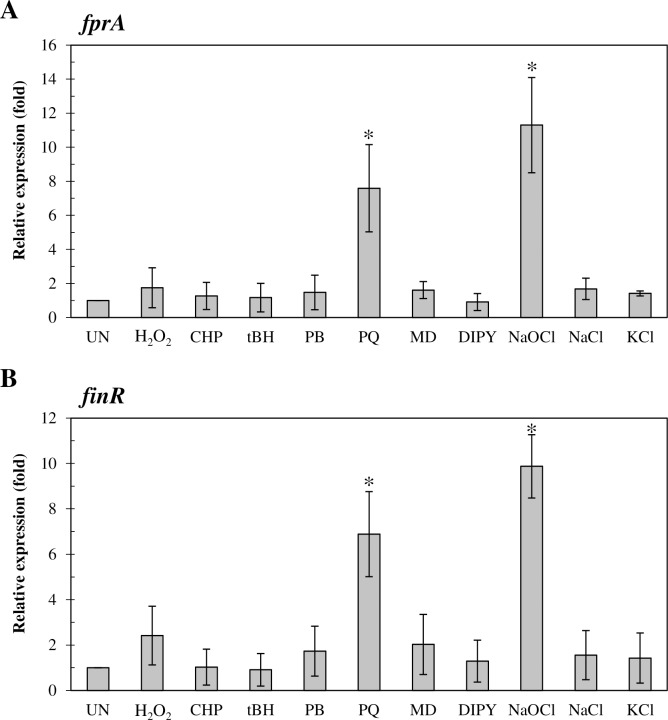
Expression analysis *finR* and *fprA* in response to various stresses. The expression levels of *finR* (A) and *fprA* (B) were determined using real-time RT-PCR. Exponential-phase cultures of *P*. *aeruginosa* PAO1 were subjected to various stress conditions, including 1 mM H_2_O_2_, superoxide anion-generating agents (0.5 mM plumbagin [PB], 0.5 mM menadione [MD] and 0.5 paraquat [PQ]), organic hydroperoxides (1 mM cumene hydroperoxide [CHP] and 1 mM *t*-butyl hydroperoxide [tBH]), 1 mM 2,2’-dipyridyl (DIPY), high salts (0.5 M NaCl and 0.5 M KCl), or 0.04% NaOCl for 15 minutes prior to RNA preparation for real-time RT-PCR analysis. Relative expression was analyzed using the 16S rRNA gene as the normalizing gene and was expressed as the fold expression relative to the level of uninduced (UN) PAO1. Data shown are means ± SD of three independent experiments.

The expression patterns of *fprA* under stressful growth conditions were evaluated using real-time RT-PCR. The expression profiles of PAO1 *fprA* challenged with superoxide anion-generating agents (plumbagin, menadione, and paraquat), H_2_O_2_, organic hydroperoxides (cumene hydroperoxide, and *t*-butyl hydroperoxide), the iron-chelating agent 2,2’-dipyridyl, high salt (NaCl and KCl), and a bleaching agent (NaOCl) were determined. The results in Fig 2A illustrate that *fprA* expression was considerably induced by exposure to paraquat (7.6 ± 2.6-fold) and NaOCl (11.3 ± 2.8-fold), but not by the other oxidants, 2,2’-dipyridyl, or high salt conditions. The expression profiles of *finR* in response to stresses were also determined by real-time RT-PCR. The expression of *finR* could be induced only by exposure to paraquat (6.9 ± 1.9-fold) or NaOCl (9.9 ±1.4-fold) treatments (Fig 2B). Other oxidants and stresses did not significantly (2-fold or less) induce *finR* expression. This induction pattern is similar to the stress response pattern of *fprA*. A previous report indicated that paraquat induction of *fprA* in *Pseudomonas* spp. is significantly affected by the addition of various sources of sulfur [31]. Nonetheless, how intracellular sulfur affects the induction of gene expression by superoxide generator is being investigated.

### FinR regulates the expression of *fprA* and itself

**Fig 3:**
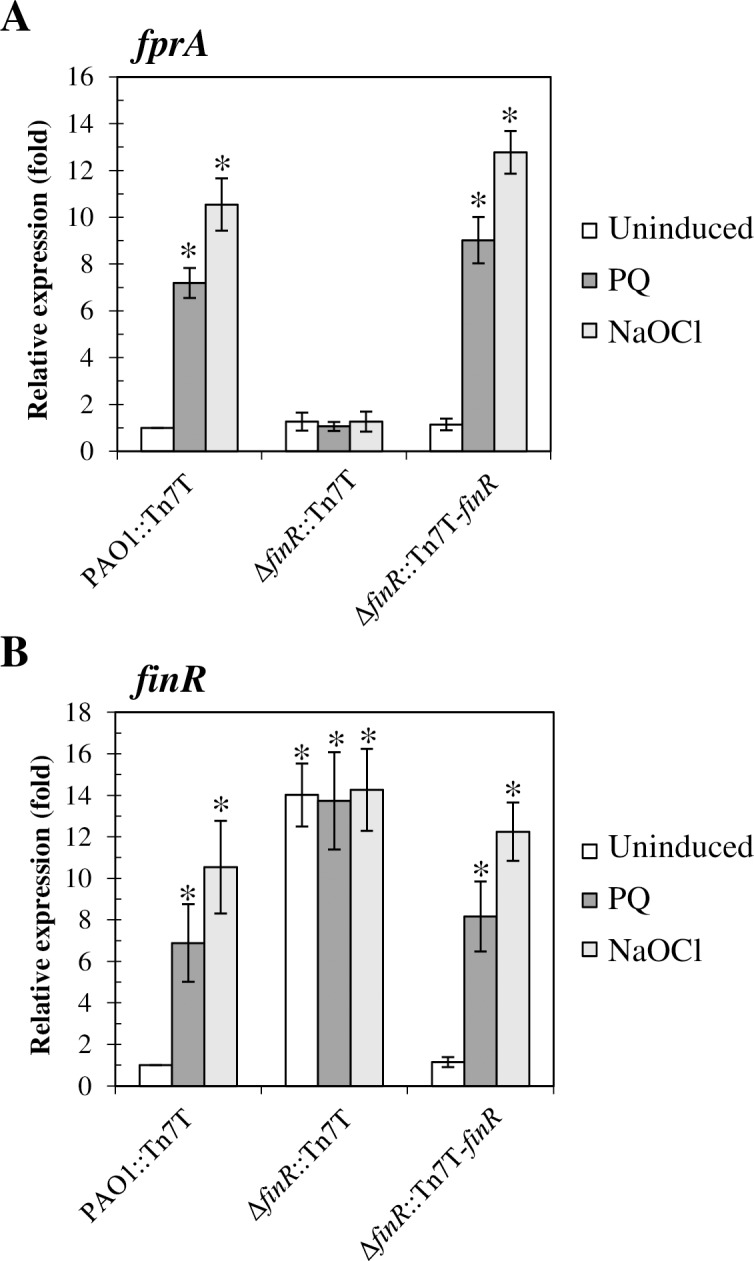
Expression analysis of *fprA* and *finR* in *P*. *aeruginosa* strains. Expression levels of *fprA* (A) and *finR* (B) in PAO1 wild type (PAO1::Tn7T), Δ*finR* mutant (Δ*finR*::Tn7T) and the complemented mutant (Δ*finR*::Tn7T-*finR*) grown under uninduced, 0.5 mM paraquat (PQ), or 0.04% NaOCl (NaOCl) induced conditions. Relative expression was analyzed using the 16S rRNA gene as the normalizing gene and is expressed as fold expression relative to the level of uninduced PAO1. Data shown are means ± SD of three independent experiments. The asterisks indicate statistically significant differences (p < 0.01) compared with the uninduced condition.

To assess whether FinR mediates induction of *fprA* expression upon exposure to oxidative stress, *fprA* expression levels were determined in the Δ*finR* mutant (Δ*finR*::Tn7T) and the complemented mutant (Δ*finR*::Tn7T-*finR*) using real-time RT-PCR. The results showed that paraquat- and NaOCl-induced expression of *fprA* was abolished in the Δ*finR* mutant and that this could be restored in the complemented mutant strain (Fig 3A). The levels of *fprA* expression in the Δ*finR* mutant in all tested conditions were comparable to those of the uninduced wild-type PAO1 (Fig 3A). Moreover, the *fprA* level in a complemented strain (Δ*finR*::Tn7T-*finR*) was comparable to wild type and a Δ*finR* mutant strain. Thus, oxidized FinR likely functions as a transcriptional activator on the *fprA* promoter in the presence of the inducers paraquat and NaOCl. However, reduced FinR neither represses nor activates *fprA* expression.

The expression levels of *finR* in response to oxidants were also evaluated in the Δ*finR* mutant and the complemented mutant using real-time RT-PCR with primers located immediately downstream of the transcriptional start site (+1) of *finR* and next to the deletion site (BT3334 and EBI62). The expression levels of *finR* in the Δ*finR* mutant were constitutively high (~14-fold over wild-type PAO1 levels) in both uninduced and oxidant-induced samples (Fig 3B). The constitutively high expression levels in the *finR* mutant strongly suggest that reduced FinR functions as a transcriptional repressor of its own promoter. Paraquat- and NaOCl-induced *finR* expression could be restored in the complemented Δ*finR* mutant strain (Δ*finR*::Tn7T-*finR*) (Fig 3B). This suggests that reduced FinR functions as a repressor of *finR* expression while oxidized FinR either activates expression or derepresses *finR* expression. The results indicate that *finR* is negatively auto-regulated, which is similar to other transcriptional regulators in the LysR-family [25, 43–45].

### FinR binds directly to *finR-fprA* promoter region

**Fig 4:**
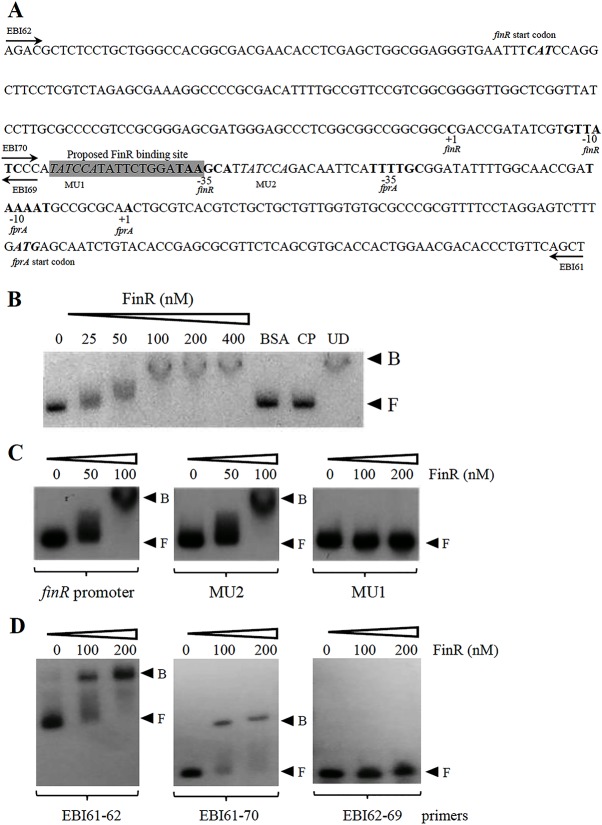
Characterization and binding of purified FinR to the *finR-fprA* promoter. (A) Nucleotide sequence showing the *finR*-*fprA* promoter structure. +1 indicates the transcriptional start site, and the bold sequences are the putative -35 and -10 promoter motifs. ***CAT*** and ***ATG*** are the translational start codons of FinR and FprA, respectively. The box shaded gray represents the proposed FinR binding site. (B), (C), and (D) Electrophoretic mobility shift assay using purified FinR. A ^32^P –labeled DNA fragment (B), mutagenized MU1 and MU2 fragments (C), or the promoter fragments (EBI61-62), with (EBI 61–70) and without (EBI 62–69) proposed FinR binding site (D) spanning the *finR-fprA* promoter was incubated with increasing amounts of FinR. BSA represents an unrelated protein (2.5 μg BSA). CP and UD signify the cold probe (100 ng unlabeled promoter fragment) and unrelated DNA (1 μg of pUC18 plasmid), respectively, that were added to the binding reaction mixture containing 100 nM FinR. F and B indicate free and bound probes, respectively.

*fprA* is located next to a divergently transcribed gene, *finR*, with a 273-bp intergenic region. To characterize the *fprA* and *finR* promoters, the putative +1 sites were determined using 5’ RACE. The +1 site of *fprA* was mapped to an A residue located 54 bp upstream of its translational start codon ATG (Fig 4A). Two sequences (TTTTGC and TAAAAT, separated by 18 bp) that resemble the *E*. *coli* δ^70^–35 and -10 promoter motifs were identified. Using a similar technique, the +1 site of *finR* was mapped to a G residue situated 125 bp upstream of the *finR* start codon (ATG) and 97 bp upstream of the putative *fprA* +1 (Fig 4A). The -35 and -10 promoter motifs were identified as TGCTTA and GATAAC and were separated by 18 bp. The *fprA* and *finR* promoter motifs did not overlap with each other (Fig 4A).

The ability of purified FinR to bind to the *fprA*-*finR* promoter was investigated using electrophoretic mobility shift assays (EMSA). A 6His-tagged FinR protein was purified using an *E*. *coli* system (25]. A [P^32^]-labeled DNA probe (398 bp) spanning the *fprA*-*finR* promoters was used in the binding experiments. Purified FinR could bind to the *fprA*-*finR* promoter sequence at nanomolar concentrations (Fig 4B). The binding specificity of FinR was illustrated by the ability of excess unlabeled *fprA*-*finR* promoter fragment (CP) but not excess of unrelated DNA (pUC18 plasmid, UP) to compete with the binding of FinR to labeled promoter fragments. Addition of an excess amount of unrelated protein (2.5 μg bovine serum albumin [BSA]) did not affect binding of purified FinR to the *fprA*-*finR* promoter (Fig 4B). Thus, FinR functions as a transcriptional regulator of *fprA* and *finR* itself through a direct binding to the *fprA*-*finR* promoter region.

To our knowledge, no consensus sequence for FinR binding box on target gene promoters has been identified. FinR is a member of LysR family of transcription regulators, which often use palindromic DNA sequences as a binding box that the regulator in LysR family binds to modulate expression of the target gene [46]. We identified DNA sequences with two overlaps and almost perfect dyadic symmetry, 5’TATCCATATTCTGGATAAGCATTATCCAGA3’, consisting of the first palindrome 5’TATCCATATTCTGGATA3’ and the second palindrome 5’TCTGGATAAGCATTATCCAGA3’ located between positions -22 and -51 of the *finR* promoter and -46 and -83 of the *fprA* promoter (Fig 4A). The involvement of these two dyadic symmetries in the binding of FinR was evaluated. Site-directed mutagenesis was performed to mutate the putative binding site for FinR from 5’TATCCATATTCTGGATAAGCATTATCCAGA3’ to 5’GCGAACTATTCTGGATAAGCATTATCCA3’ (referred to as MU1) and to 5’TATCCATATTCTGGATAAGCATGCGAACGA3’ (referred to as MU2) using pP_*fprA*_ as a DNA template. The mutations essentially changed the first palindrome sequence in MU1 and the second palindrome sequence in MU2. [P^32^]-labeled *fprA-finR* promoter containing MU1 or MU2 sequences was used in the EMSA experiments. The results in Fig 4C showed that purified FinR bound to the promoter containing MU2 in a similar manner as the native promoter. However, purified FinR at concentration of 200 nM was unable to bind the mutagenized MU1 promoter (Fig 4C). This suggests that the sequence TATCCA of the first palindromic sequence 5’TATCCATATTCTGGATA3’ is important for in vitro binding of *P*. *aeruginosa* FinR. To confirm the putative binding site of FinR, the EMSA experiments were performed using the promoter fragment with and without proposed FinR binding site. The results in Fig 4D showed that the purified FinR could bind to the promoter fragment with the proposed FinR-binding site. No FinR binding could be detected when the DNA fragment without the binding site was used (Fig 4D). This supports the site-directed mutagenesis results that in vitro FinR binds specifically to the palindromic sequence 5’TATCCATATTCTGGATA3’.

### Δ*finR* mutant shows an increased paraquat sensitivity phenotype that could be suppressed by increasing *fprA* expression

**Fig 5:**
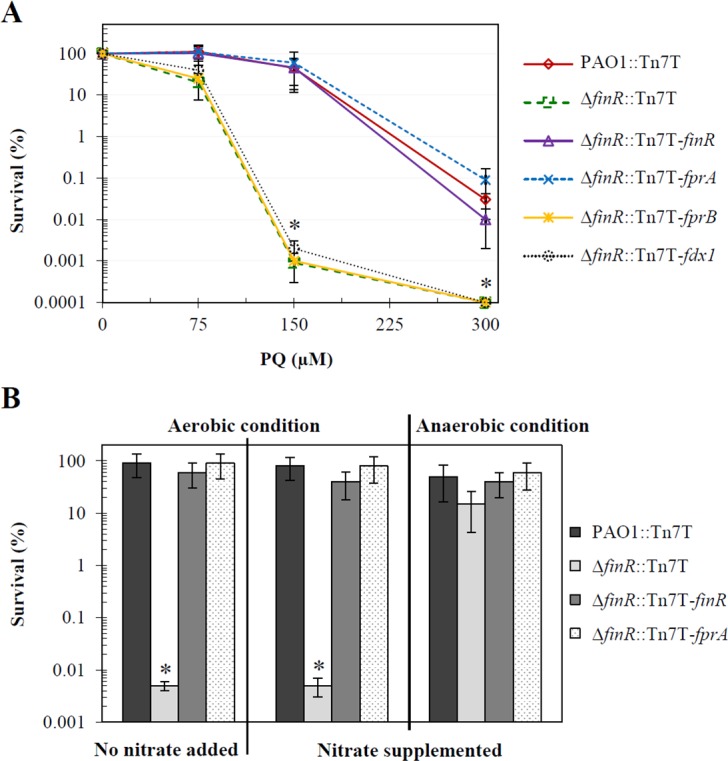
Determination of paraquat resistance levels in *P*. *aeruginosa* strains. (A) Paraquat resistance levels in PAO1 containing the mnin-Tn7 vector control (PAO1::Tn7T, red) and Δ*finR* mutants containing Tn7T (dotted green), Tn7T-*finR* (purple), Tn7T-*fprA* (dotted blue), Tn7T-*fprB* (yellow), or Tn7T-*fdx1* (dotted black) were determined using plate sensitivity assays. (B) Paraquat (150 μM) resistance levels of *P*. *aeruginosa* strains were determined using LB with and without 1% (w/v) KNO_3_ supplementation and incubated under aerobic and anaerobic atmospheres. The survival is expressed as a percentage of the CFU on LB plates containing paraquat over the CFU on plates without paraquat. Data shown are means ± SD from three independent experiments.

Next, the physiological function of *finR* was assessed using the Δ*finR* mutant. Since FinR is involved in sensing various oxidant resistance levels, the Δ*finR* mutant resistance to oxidants was determined using a plate sensitivity assay. The Δ*finR* mutant exhibited similar levels of resistance to various oxidants, including H_2_O_2_, cumene hydroperoxide, and NaOCl, as the wild-type PAO1 (data not shown). Nonetheless, Fig 5A shows that the Δ*finR* mutant (Δ*finR*::Tn7T) was much more sensitive (10^4^-fold) to paraquat (150 μM) than its parental strain PAO1 (PAO1::Tn7T), and this hypersensitive phenotype of the mutant was fully restored by the expression of a single copy of functional *finR* in a mini-Tn7 vector (Δ*finR*::Tn7T-*finR*). These results indicate a crucial role of *finR* for survival under paraquat stress and are consistent with the previously reported resistance of a *finR* mutant of *P*. *putida* [25].

Paraquat is a redox cycling drug that has been recognized as a superoxide anion-generating agent in the presence of oxygen by disrupting normal electron flow in aerobic respiration [17]. The drug itself can undergo intracellular transformations and is toxic to cells [19]. The question was raised as to whether hypersensitivity of the *finR* mutant to paraquat was due to reduced ability to detoxify superoxide anions generated from the drug or direct toxicity of the drug. An approach previously described in *E*. *coli* was used to test the likely mechanism responsible for paraquat sensitivity in the Δ*finR* mutant; this approach used anaerobic cultivation to distinguish between the direct toxicity of the drug and the generation of superoxide anions, which requires oxygen [19]. *P*. *aeruginosa* did not grow under anaerobic conditions unless nitrate was added to the culture medium [47]. Plate sensitivity assays were performed to re-examine the paraquat sensitivity levels using LB medium supplemented with potassium nitrate (KNO_3_, 1% w/v) under anaerobic conditions. All *P*. *aeruginosa* grew anaerobically (data not shown). The results of the paraquat sensitivity assay under aerobic and anaerobic conditions are shown in Fig 5B. The Δ*finR* mutant (Δ*finR*::Tn7T) was much more sensitive (10^4^-fold) to paraquat (150 μM) under aerobic growth than the parental PAO1 (PAO1::Tn7T), whereas no significant change was observed when the plates were incubated under anaerobic conditions. Thus, the killing effects of paraquat are oxygen-dependent and likely occur by generating superoxide anions. Hence, the observed increased sensitivity to paraquat in the Δ*finR* mutant most likely is a result of superoxide killing. PAO1 produces two superoxide dismutase (Sod) isozymes, namely SodA (manganese-containing Sod) and SodB (iron-containing Sod); mutations of either *sodA* or *sodB* enhance sensitivity to superoxide anions generated from paraquat [48]. We tested whether the paraquat-sensitive phenotype of the *finR* mutant was due to lower level of Sod activity; total Sod activity was measured in the *finR* mutant cultivated aerobically. The results showed non-significant differences in the levels of total Sod activity in the *finR* mutant relative to wild-type PAO1 (data not shown). Therefore, alterations in levels of paraquat resistance of the *finR* mutant are independent of total Sod enzyme activity.

We have shown that *fdx1* could suppress the Δ*fprA* essentiality phenotype. Hence, we tested whether expression of *fprB* or the ferredoxin-encoding genes *fdx1*, *fdx2*, *fdxA* and *rnfB* could complement the paraquat hypersensitivity phenotype of the Δ*finR* mutant, and the results showed that expression of these genes could not complement the *finR* mutant phenotype (data not shown). Here, we have established that FinR positively regulates *fprA* expression, and therefore, we speculate that the paraquat hypersensitive phenotype of the Δ*finR* mutant could arise from loss of the ability to activate *fprA* expression upon exposure to paraquat. Expression of *fprA* under the control of the *lac* promoter in a mini-Tn7 vector was transposed into the Δ*finR* mutant, generating Δ*finR*::Tn7T-*fprA*. The paraquat resistance levels of this strain were evaluated. The results in Fig 5A illustrate that increased expression of *fprA* completely restored the paraquat sensitivity of the Δ*finR* mutant to the levels that were attained by the Δ*finR*::Tn7T-*finR* and a wild-type control (PAO1::Tn7T). Since basal levels of *fprA* expression in the Δ*finR* mutant and the parental strain are similar (Fig 3A), the results suggest that the paraquat-hypersensitive phenotype of the Δ*finR* mutant could be due to the inability of the mutant to up-regulate the expression of *fprA* in response to stressful conditions. This suggests that the levels of FprA are critically important, especially under certain stress conditions (i.e., paraquat and NaOCl).

### Δ*finR* mutant shows attenuated virulence in a Drosophila host model

**Fig 6:**
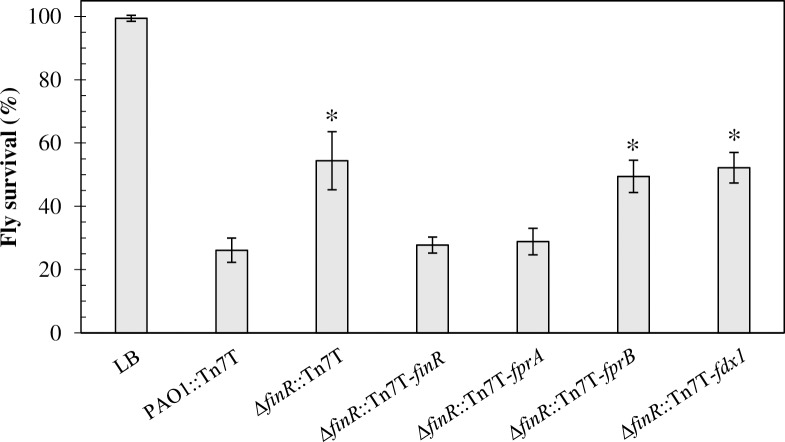
Virulence of *P*. *aeruginosa* strains. The virulence of PAO1 containing the Tn7T vector control (PAO1::Tn7T) and Δ*finR* mutants containing Tn7T, Tn7T-*finR*, Tn7T-*fprA*, Tn7T-*fprB*, or Tn7T-*fdx1* were determined using the *Drosophila melanogaster* feeding method. The percent fly survival was scored after 18 hours of incubation. Data presented are means ± SD of three independent experiments. The asterisk indicates statistically significant difference (p < 0.01) compared with PAO1::Tn7T. LB represents feeding the flies with LB medium.

FinR positively regulates the expression of an essential gene, *fprA*, in response to oxidative stress; therefore, the contribution of *finR* to the bacterial pathogenicity of *P*. *aeruginosa* was evaluated using the fruit fly (*Drosophila melanogaster*) as a pathogen-host model as previously described [5, 49]. As shown in Fig 6, feeding the flies with PAO1 cultures resulted in 26.1 ± 3.9% fly survival compared with 99.4 ± 1.0% fly survival when LB medium was fed to the flies as a negative control. Feeding the flies with Δ*finR* mutant cultures resulted in a 2-fold increase in fly survival (54.4 ± 9.2%) compared with feeding with PAO1. Thus, deletion of *finR* attenuated the virulence of *P*. *aeruginosa* PAO1 in the tested model (p < 0.01). The attenuated virulence phenotype of the Δ*finR* mutant could be restored in the complemented mutant strain (Δ*finR*::Tn7T-*finR*), which expressed a functional copy of *finR* (27.8 ± 2.6% fly survival). Additionally, expressing *fprA* could complement the attenuated virulence phenotype of the *finR* mutant (Δ*finR*::Tn7T-*fprA*), as shown by 28.9 ± 4.2% fly survival, while expressing *fprB* (Δ*finR*::Tn7T-*fprB*) could not (49.4 ±5.1% fly survival) (Fig 6). The phenotype of attenuated virulence was consistent with that of paraquat sensitivity levels, in which expression of *fprA* restored the Δ*finR* mutant phenotype (Fig 5A). The facts that increased expression of *fprA* could restore the Δ*finR* mutant phenotype and that basal expression of *fprA* (Fig 4A) in the Δ*finR* mutant was comparable to that of the PAO1 wild type suggested that loss of adaptive expression of *fprA*, which is modulated by FinR, is responsible for the virulence attenuation as well as the paraquat hypersensitivity of the Δ*finR* mutant. We also present here that the paraquat-sensitive phenotype of the Δ*finR* mutant involved superoxide anion-mediated toxicity (Fig 5B). In several plant and animal pathogenic bacteria, defects in superoxide anion detoxification systems, such as knockout of superoxide dismutase genes, render the mutant strains attenuated for virulence in the model hosts [50–52]. Superoxide anions are one of the key components of innate immunity generated by host cells to eradicate invading microbes. In human hosts, superoxide anions are produced within the phagolysosomes of phagocytic cells to kill the engulfed pathogens [53]. Thus, defects in protection against superoxide toxicity of the bacteria would reduce the ability to survive within the host. Hence, the attenuated phenotype could result from the reduced ability of the Δ*finR* mutant to cope with exposure to superoxides during host interactions.

Alternatively, in pseudomonads, FprA plays a role in sulfur metabolism and cysteine biosynthesis, which are important components of [Fe-S] cluster biogenesis [31, 54]. [Fe-S] clusters, which are key cofactors of proteins that are implicated in diverse cellular processes, including respiration and central metabolism, are prone to oxidative damage when cells are exposed to reactive oxygen species (ROS) such as superoxide anions and H_2_O_2_ [54, 55]. Therefore, impaired [Fe-S] cluster biogenesis during exposure to oxidative stress due to lack of FinR-mediated increased expression of *fprA* would lead to lowered ability of the bacteria to survive oxidative stress generated by the host’s innate immune system. Similar mechanisms could also account for the observed paraquat hypersensitive phenotype. Mutants defective in [Fe-S] cluster biogenesis or repair, for example, deletion of the IscR coding gene, which regulates [Fe-S] cluster biogenesis, show attenuated virulence in host models, including such mutants of *P*. *aeruginosa* [56–59].

Since *fprA* is essential in PAO1, a direct analysis of the mutant phenotypes is difficult. Analysis of the Δ*finR* mutant provides insight into the importance of *fprA*. Δ*finR* mutant phenotypes (paraquat sensitivity and attenuated virulence) most likely occur as a result of the inability of *fprA* to be up-regulated during stressful conditions. This suggests that the level of FprA is crucial in *P*. *aeruginosa*. An *fdx1* encoding putative reaction partner of FprA is also an essential gene in PAO1 [40]. Thus, the link between FprA and Fdx1 is important to PAO1 physiology.

## Materials and methods

### Bacterial strains, plasmids and growth conditions

Both *E*. *coli* and *P*. *aeruginosa* (PAO1, ATCC15692) strains were aerobically cultivated in Luria-Bertani (LB) broth (BD Difco, USA) at 37°C with shaking at 180 rpm unless otherwise stated. To produce synchronous growth, an overnight culture was inoculated into fresh LB medium to give an optical density at 600 nm (OD_600_) of 0.1. Exponential phase cells (OD_600_ of about 0.6, after 3 h of growth) were used in all experiments. All plasmids used in this study are listed in Table 2.

**Table 2:** List of plasmids used in this study.

Plasmid	Relevant characteristic(s)	Source or Reference
pBBR1MCS-4	Broad-host-range expression vector, Ap^r^	[37]
pSS255	Expression vector with a temperature sensitive replicon (mSF^*ts1*^), Ap^r^	[36]
pTS	pBBR1MCS-4 carrying mSF^*ts1*^, Ap^r^	This study
pTS-*fprA*	pTS carrying *fprA*	This study
pTS-*fprB*	pTS carrying *fprB*	This study
pTS-*fdx1*	pTS carrying *fdx1*	This study
pTS-*fdx2*	pTS carrying *fdx2*	This study
pTS-*rnfB*	pTS carrying *rnfB*	This study
pTS-*fdxA*	pTS carrying *fdxA*	This study
pUCΔ*finR*::Gm^r^	pUC18 containing Gm^r^ inserted into deleted *finR*, Gm^r^	This study
pUCΔ*fprA*::Gm^r^	pUC18 containing Gm^r^ inserted into deleted *fprA*, Gm^r^	This study
pCM351	vector containing the *loxP*-Gm^r^-*loxP* region, Gm^r^	[61]
pCM157	vector containing the Cre-encoding gene, Tet^r^	[61]
pUC18-mini-Tn7T::Gm-LAC	mini-Tn7 vector with P_tac_ expression cassette, Gm^r^	[34]
pTNS2	Helper plasmid for Tn7 insertion, Ap^r^	[34]
pTn-*finR*	pUC18-mini-TN7T::Gm-LAC containing *finR*	This study
pTn-*fprA*	pUC18-mini-TN7T::Gm-LAC containing *fprA*	This study
pTn-*fprB*	pUC18-mini-TN7T::Gm-LAC containing *fprB*	This study
pTn-*fdx1*	pUC18-mini-TN7T::Gm-LAC containing *fdx1*	This study
pTn-*fdx2*	pUC18-mini-TN7T::Gm-LAC containing *fdx2*	This study
pTn-*rnfB*	pUC18-mini-TN7T::Gm-LAC containing *rnfB*	This study
pTn-*fdxA*	pUC18-mini-TN7T::Gm-LAC containing *fdxA*	This study
pP_*fprA*_	pUC18 carrying *fprA* promoter	This study
pP_*fprA*_MU1	pUC18 carrying mutagenized *fprA* promoter MU1	This study
pP_*fprA*_MU2	pUC18 carrying mutagenized *fprA* promoter MU2	This study
pQE30Xa	Vector for expressing N-terminal 6His tagged protein in *E*. *coli*, Ap^r^, Cm^r^	Qiagen (Germany)
pQE30Xa-*finR*	pQE30XA carrying full-length *finR*	This study

Gm^r^, gentamicin resistance; Ap^r^, ampicillin resistance; Tet^r^, tetracycline resistance; Cm^r^ chloramphenicol resistance.

### Molecular techniques

General molecular techniques including DNA and RNA preparations, DNA cloning, PCR amplification, Southern analyses and bacterial transformation were performed according to standard protocols [60]. The oligonucleotide primers used in this study are listed in Table 3.

**Table 3:** List of primers used in this study.

**Name**	**Sequence 5’→3’**	**Purpose**
BT2781	GCCCGCACAAGCGGTGGAG	Forward primer for 16S rRNA
BT2782	ACGTCATCCCCACCTTCCT	Reverse primer for 16S rRNA
BT3332	ACGTGCACAACACCGCCC	Forward primer for full-length *finR*
BT3333	CAGGCGGATGTTCAGCGG	Reverse primer for full-length *finR*
BT3334	TAGACGAGGAAGCCTGGATG	Forward primer for *finR* fragment
BT3335	TGTCCCTGGCCAACTGAG	Reverse primer for *finR* fragment
BT3336	GGAGTTCTTCAGCATCAAGG	Forward primer for full-length *fprA*
BT3337	GAAGTACTCGTGTTCCGGCA	Reverse primer for full-length *fprA*
BT3456	GTCTGCTGCTGTTGGTGTG	Forward primer for *fprA* expression
BT3457	GGCAGGGGCTTTCTTCG	Reverse primer for *fprA* expression
BT4443	GTGGCTGTCCGTCGCGGTTG	Forward primer for full-length *fdx1*
BT4444	CAGGCGCCGGCGGGGATCAG	Reverse primer for full-length *fdx1*
BT4479	CCTTGATGCTGAAGAACTCC	Sp2 primer for *fprA*
BT4780	GCAAAATGAATTGTCGTTCGCATGCTTAT	Forward primer for mutated *finR* promoter MU2
BT4781	CTGGATAAGCATGCGAACGACAATTC	Reverse primer for mutated *finR* promoter MU2
BT4782	CTTATCCAGAATAGTTCGCTGGGATAA	Forward primer for mutated *finR* promoter MU1
BT4783	CGTGTTATCCCAGCGAACTATTCTGG	Reverse primer for mutated *finR* promoter MU1
BT3499	GTGCTTTGCGGGACACTAGG	Forward primer for full-length *fprB*
BT3500	GCTATCCGCCGCTACTGC	Reverse primer for full-length *fprB*
BT5019	CCTGGGCGGTGTTGTGCA	Sp1 primer for *finR*
BT5201	GAGGAGAGAACTAGAAAATG	Forward primer for full-length *fdxA*
BT5309	CTTGGCGTATCAGCGCTC	Reverse primer for full-length *fdxA*
EBI01	CATGGGCTTCAGCGGGTTGG	Forward primer for full-length *rnfB*
EBI02	GTGCAGGGCGCTCATGCC	Reverse primer for full-length *rnfB*
EBI53	GGGAATTCGAAGTACTCGTGTTCCGGCA	Forward primer for upstream fragment of *finR*
EBI54	GGCCATGGGAACAGCTTGCAGTCGAACTG	Reverse primer for upstream fragment of *finR*
EBI57	CCGAATTCTCCAGCTCGTAGTGGGCGAC	Forward primer for upstream fragment of *fprA*
EBI58	GGCCATGGTAGTTCGGGCTGGCAATGCTG	Reverse primer for upstream fragment of *fprA*
EBI61	AGCTGAACAGGGTGTCGT	Forward primer for *finR* promoter
EBI62	AGACGCTCTCCTGCTGGG	Reverse primer for *finR* promoter
EBI69	GGGATAACACGATATCGGTCGG	Forward primer for *finR* promoter
EBI70	CGATATCGTGTTATCCCATATCC	Reverse primer for *finR* promoter
EBI73	CCATCGATCGATCAAGCGTGCCGTGGAG	Forward primer for downstream fragment of *finR*
EBI74	CCGGAGCTCTGCTGCTGGGGATCGTCCTG	Reverse primer for downstream fragment of *finR*
EBI75	CCATCGATGGCAAGCTGTTCGAGGACATC	Forward primer for downstream fragment of *fprA*
EBI76	CCGGAGCTCCCTCAGCCAGGGTCACCTGAGC	Reverse primer for downstream fragment of *fprA*
EBI269	GAACTGTCGAGGAATAAGCGAAGATGCC	Forward primer for full-length *fdx2*
EBI270	ATTGCACGCTCCTCTACTAC	Reverse primer for full-length *fdx2*
EBI292	GCGCCTGCAGTCAGGGAATCAGCGGCA	Reverse primer for FinR protein expression
EBI322	ATGAAATTCACCCTCCGC	Forward primer for FinR protein expression

### Construction of *P*. *aeruginosa* Δ*finR* mutant

The *finR* deletion mutant was constructed using homologous recombination with an unmarked Cre-*loxP* antibiotic marker system as previously described [61]. The primer pairs, EBI73-EBI74 and EBI53-EBI54, were designed to amplify a *finR* fragment containing the C-terminus and N-terminus, respectively, of the *finR* coding region, plus additional flanking regions from the PAO1 genomic DNA. The 1030-bp PCR fragment of the C-terminus was digested with ClaI and SacI and cloned into pUC18Gm^r^ (pUC18 containing *loxP*-flanked Gm^r^, which was constructed by inserting SacI-EcoRI fragments containing *loxP*-flanked Gm^r^ from pCM351 [61] into pUC18 cut with the same enzymes) at the ClaI and SacI sites, yielding pUC*finR*C::Gm^r^. The 926-bp PCR fragment of the N-terminus was digested with EcoRI and NcoI and cloned into pUC*finR*C::Gm^r^ at the EcoRI and NcoI sites, yielding pUCΔ*finR*::Gm^r^. The constructed plasmid resulted in the deletion of 526 bp of the coding region of *finR*. pUCΔ*finR*::Gm^r^ was transferred into PAO1, and the putative Δ*finR* mutants that arose from a double crossover event were selected for the Gm^r^ and Cb^s^ phenotypes. An unmarked Δ*finR* mutant was created using the Cre-*loxP* system to excise the Gm^r^ gene as previously described [61], and deletion of *finR* was confirmed by Southern blot analysis.

### Construction of the *P*. *aeruginosa* Δ*fprA* mutant

The *fprA* deletion mutant was constructed using homologous recombination with an unmarked Cre-*loxP* antibiotic marker system using the same protocol as the construction of the Δ*finR* mutant but using primer pairs, EBI75-EBI76 and EBI57-EBI58, which were designed to amplify the *fprA* fragment containing the *fprA* coding region plus additional flanking regions. The restriction enzyme sites and plasmids were same as those used in the construction of the Δ*finR* mutant. The obtained plasmid, pUCΔ*fprA*::Gm^r^, was used to transform PAO1 wild type and strains containing either an expression plasmid or a temperature-sensitive expression plasmid. PAO1 strains containing an extra copy of various genes (*fdx*, *fdxA*, *fdx2*, *rnfB*, *finR*, *fprA*, *fprB*) was used to test the essentiality of the *fprA* gene and were constructed by transposition of a mini-Tn7 vector containing a target gene into the PAO1 chromosome and the subsequent removal of the Gm^r^ antibiotic resistance marker gene of mini-Tn7 using the Flp-FRT recombinase as previously described [35].

### Construction of plasmid and mini-Tn7 harboring gene coding regions and promoters

To construct pTS, a temperature-sensitive replicon cassette-containing plasmid, a broad-host-range plasmid pBBR1MCS-4 [37] was inserted with a BamHI fragment containing the temperature-sensitive (TS) regulon isolated from vector pTS225 [36] at the BamHI site. pTS-*fprA* was constructed by amplifying the full-length *fprA* from the PAO1 genomic DNA with primers BT3456-BT3457. The 866-bp PCR products were cloned into the pTS cut with SmaI. A similar protocol was used to construct pTS-*fdx1*, pTS-*fdx2*, pTS-*fdxA*, pTS-*rnfB* and pTS-*fprB* for *trans* expression of *fdx1* (PA0362), *fdx2* (PA3809), *fdxA* (PA3621), *rnfB* (PA3490) and *fprB*, respectively. The specific primer pairs for PCR amplification of full-length *fdx1*, *fdx2*, *fdxA*, *rnfB*, and *fprB* genes were BT4443-BT4444, EBI269-EBI270, BT5201-BT5309, EBI01-EBI02 and BT3499-BT3500, respectively.

Single-copy complementation was performed using a mini-Tn7 system [34]. The full-length PCR fragments of various genes were PCR amplified with specific primer pairs as described above (and BT3334-BT3335 for *finR*) and cloned into pUC18-mini-Tn7T-Gm-LAC [34], generating *pTn-fprA*, pTn-*fdx1*, pTn-*fdx2*, pTn-*fdxA*, pTn-*rnfB*, pTn-*fprB* and pTn-*finR*. The mini-Tn based recombinant plasmid were then transposed into either PAO1 or mutant strains, generating the complemented strains Δ*finR*::Tn7T-*finR* and Δ*finR*::Tn7T-*fprA*. Confirmation of transposition was carried out as previously described [34].

To construct the plasmids containing the *fprA* promoter region, a putative *fprA* promoter fragment was amplified from the PAO1 genomic DNA with primers EBI61 and EBI62. The 398-bp PCR product was ligated into EcoRV-digested pUC18 and was named pP_*fprA*_. PCR-based site-directed mutagenesis at the putative FinR-binding site was performed as previously described [6] using primers BT4782-BT4783 and BT4780-BT4781, and these vectors are referred to as pP_*fprA*_-MU1 and pP_*fprA*_-MU2, respectively.

### 5’ rapid amplification of cDNA ends (RACE)

5’ RACE was performed using a 5’/3’ RACE kit (Roche, Germany) as previously described [62]. Essentially, DNase I-treated total RNA was reverse transcribed using specific primers BT3311 and BT3337 as SP1 primers for *finR* and *fprA*, respectively. The first-strand DNA (cDNA) was purified, and poly(A) was added to the 5’-terminus of the cDNA using terminal transferase. Next, poly(A)-tailed cDNA was PCR-amplified using the specific SP2 primer BT4438 for *finR* and BT4479 for *fprA* and an anchored oligo(dT) primer. The purified PCR product was cloned into the pGemT vector, and the +1 site was identified from the DNA sequences.

### Real-time RT-PCR

Reverse transcription was performed as described for end-point RT-PCR [63]. Real-time RT-PCR was conducted using 10 ng of cDNA as template, a specific primer pair and a KAPA SYBR® FAST qPCR kit (Kapa Biosystems, USA). The reaction was run on an Applied Biosystems StepOnePlus thermal cycler under the following conditions: denaturation at 95°C for 20 s, annealing at 60°C for 30 s, and extension at 60°C for 30 s, for 40 cycles. The specific primer pairs used for *finR* and *fprA* were BT3334-EBI69 and BT3336-BT3337, respectively. The primer pair for the 16S rRNA gene was BT2781-BT2782, which was used as the normalizing gene. Relative expression analysis was calculated using StepOne software version 2.1 and is presented as expression fold-change relative to the level of PAO1 wild type grown under uninduced conditions. Experiments were repeated independently three times, and the data shown are the means with standard deviations (SD).

### Expression and purification of *P*. *aeruginosa* FinR

The 6His-tagged FinR from *P*. *aeruginosa* was purified using the pQE-30Xa expression system (Qiagen, Germany). The full-length *finR* gene was amplified from PAO1 genomic DNA with primers EBI322 and EBI292. A 937-bp PCR product was digested with PstI before ligation into pQE-30Xa digested with StuI (blunt ended) and PstI to generate pQE30Xa-*finR* for high-level expression of *finR* containing an N-terminal 6His-tag. An *E*. *coli* DH5α strain harboring pQE30Xa-*finR* was grown in LB medium containing 100 μg/ml ampicillin at 37°C to an OD_600_ of 1.0 before being induced with 100 μM IPTG for 60 min. Purification of 6His-tagged FinR was carried out using a nickel-nitrilotriacetic acid (Ni-NTA) agarose column as previously described [63]. The purity of the FinR protein was more than 90%, as judged by a major band corresponding to the 36.9-kDa protein observed on SDS-PAGE.

### Gel mobility shift assay

Gel mobility shift assays were performed using a labeled probe containing either native or mutagenized *fprA*-promoters amplified from pP_*fprA*_, pP_*fprA*_-MU1, or pP_*fprA*_-MU2 as a template and using ^32^P-labeled BT4691 and BT4692 primers. The promoter fragments (EBI61and EBI62) with and without proposed FinR binding site were amplified from genomic PAO1 using primers, EBI61-EBI70 and EBI62-EBI69, respectively. Binding reactions were conducted using 3 fmol of labeled probe in 25 μl of reaction buffer containing 20 mM Tris-HCl (pH 8.0), 50 mM KCl, 4 mM MgCl_2_, 0.5 mM EDTA, 0.02 mg ml^-1^ bovine serum albumin (BSA), 5 mM dithiothreitol (DTT), 10% (v/v) glycerol, and 200 ng of poly(dI-dC). Various amounts of purified FinR were added, and the reaction mixture was incubated at 25°C for 20 min. Protein-DNA complexes were separated by electrophoresis on a 5% non-denaturing polyacrylamide gel in 0.5× Tris-borate-EDTA buffer at 4°C and were visualized by exposure to Hyperfilm (GE Healthcare).

### Plate sensitivity assay

A plate sensitivity assay was performed to determine the oxidant resistance level as previously described [63]. Briefly, exponential phase cells were adjusted to OD_600_ of 0.1 before making 10-fold serial dilutions. 10 μl of each dilution was then spotted onto LB agar plate containing appropriate concentrations of testing reagents. The plates were incubated overnight at 37°C before the colony forming units (CFU) were scored. Percent survival was defined as the CFU on plates containing oxidant divided by the CFU on plates without oxidant and multiply by 100.

### Drosophila virulence tests

The virulence of *P*. *aeruginosa* was investigated using the *Drosophila melanogaster* feeding assay as previously described [5]. Briefly, exponential phase cultures of each *P*. *aeruginosa* strains were adjusted to an OD_600_ of 0.5 before 800 μL of the bacterial cells were overlaid to completely cover the surface of the preservative-free corn flour *Drosophila* medium at the bottom of a glass fly culture vial. Approximately one-week-old adult flies were starved for 3 h prior to the feeding assay. Twenty flies were added to each vial, and each strain of *P*. *aeruginosa* was tested for at least three replications. Then, all of the tested flies were incubated at 25°C for 18 h before the number of the viable flies was observed. The experiments were performed in a double-blind fashion and were analyzed from nine experiments using three different batches of flies.

### Statistical analysis

The significance of differences between strains, cultured conditions, or changes of expression level was statistically determined using Student’s t-test. P < 0.05 is considered significant difference and indicated as an asterisk.

## Supporting information

S1 Fig Multiple amino acid sequence alignment of *P*. *aeruginosa* FprA and FinR. The deduced amino acid sequence of *P*. *aeruginosa* (A) FprA ferredoxin NADP(+) reductase A and (B) FinR transcriptional regulator was aligned with those in *Pseudomonas putida* and *Azotobacter vinelandii* by using CLASTAL Omega alignment. The asterisk, colon, and period symbols indicate identical residues, conserved substitutions, and semi-conserved substitutions, respectively.
(PDF)
