# Supplementary material for: The FinR-regulated essential gene fprA, encoding ferredoxin NADP+ reductase: Roles in superoxide-mediated stress protection and virulence of Pseudomonas aeruginosa
Source: PLoS One. 2017 Feb 10;12(2):e0172071. doi: 10.1371/journal.pone.0172071 (PMC5302815; doi:10.1371/journal.pone.0172071)
Supplement: S1 Fig — The deduced amino acid sequence of P. aeruginosa (A) FprA ferredoxin NADP(+) reductase A and (B) FinR transcriptional regulator was aligned with those in Pseudomonas putida and Azotobacter vinelandii by using CLASTAL Omega alignment. The asterisk, colon, and period symbols indicate identical residues, conserved substitutions, and semi-conserved substitutions, respectively. (PDF) [file pone.0172071.s001.pdf]

A FprA

```
Pae    MSNLYTERVLSVHHWNDTLFSFKTTRNPGLRFKTGQFVMIGLEVD-GRPLMRAYSIASPN
Ppu    MSNMNHERVLSVHHWNDTLFSFKCTRDPGLRFENGQFVMIGLQQDNGRPLMRAYSIASPN
Avi    MSNLNVERVLSVHHWNDTLFSFKTTRNPSLRFENGQFVMIGLEVD-GRPLMRAYSIASPN
       ***:  ***************** **:*.***:.********: * **************

Pae    YEEHLEFFSIKVPDGPLTSRLQHLKEGDELMVSRKPTGTLVHDDLLPGKHLYLLSTGTGM
Ppu    WEEHLEFFSIKVPDGPLTSQLQHLKEGDEIIISKKPTGTLVLDDLNPGKHLYLLSTGTGL
Avi    YEEHLEFFSIKVQNGPLTSRLQHLKEGDELMVSRKPTGTLVTSDLLPGKHLYMLSTGTGL
       :*********** :*****:*********:::*:******* .** ******:******:

Pae    APFLSVIQDPETYERYEKVILVHGVRWVSELAYADFITKVLPEHEYFGDQVKEKLIYYPL
Ppu    APFMSVIQDPETYERFEKVILVHGVRYVNEVAYREFITEHLPQNEFFGESVRDKLIYYPT
Avi    APFMSLIQDPEVYERFEKVVLIHGVRQVNELAYQQFITEHLPQSEYFGEAVKEKLIYYPT
       ***:*:*****.***:***:*:**** *.*:** :***: **: *:**: *::******

Pae    VTREPFRNQGRQTDLMRSGKLFEDIGLPPMNPQDDRAMICGSPSMLEETSAVLDSFGLKI
Ppu    VTREPFENQGRLTDLMRSGKLFSDIGLPPINPQDDRAMICGSPSMLDETSEVLDSFGLKI
Avi    VTRESFHNQGRLTDLMRSGKLFEDIGLPPINPQDDRAMICGSPSMLDESCEVLDGFGLKI
       ****.*.**** **********.******:****************:*:. ***.*****

Pae    SPRMGEPGDYLIERAFVEK
Ppu    SPRMREPGDYLIERAFVEK
Avi    SPRMGEPGDYLIERAFVEK
       **** **************
```

B FinR

```
Pae    MKFTLRQLEVFVAVAQQESVSRAAEGLSLSQSATSTSLGELERQFDCKLFDRAGKRLTLN
Ppu    MRFTLRQMQVFVAVAQHQSVSRAASLLALSQSAASTSITELERQSSCQLFDRAGKRLALN
Avi    MRFTLRQLEVFAAVARLESVTRAAESLSLSQSAASTSLTELERQSDCQLFDRVGKRLKLN
       *:*****::**.***: :**:***. *:*****:***: ***** .*:****.**** **

Pae    ALGRQLLPQAVALLDRGEEIENLLNGKTAFGSLDLGATLTIGNYLATLLIGAFMQRQPDC
Ppu    ALGHQLLPQAVALLDQAKEIEDLLNGKSGFGSLAVGATLTIGNYLATLLIGNYMQTHPES
Avi    ALGRQLLPQAVALLNQAREIENLLDGKEGFGSLAVGATLTVGNYLATLLIGAFMQRHPES
       ***:**********::..***:**:** .**** :*****:********** :** :*:.

Pae    RVRLHVHNTAQVVQQVAHYELDLGLIEGDCRHPDIEVQPWMEDELVVFCAPQHPLARRGG
Ppu    QVKLHVQNTAHIVQQVAHYEIDLGLIEGDCNHPDLEVQPWVEDELVVFCAPQHPLAKLGR
Avi    RVRLHVQNTAHIVQQVVNHELDLGLIEGNCQHPDVELQPWVEDELVVFCAPQHPLARHGR
       :*:***:***::****.::*:*******:*.***:*:***:***************: *

Pae    ADLERLTREAWILREQGSGTRLTFDQAMRHHPRPLNIRLELEHTEAIKRAVESGLGIGCI
Ppu    ADIESLSQEAWILREQGSGTRLTFDQAMRHHRANLNIRLELEHTEAIKRAVESGLGIGCI
Avi    ATLDELAGEAWILREQGSGTRLTFDQAMRHHRPMLNIRLELEHTEAIKRAVESSLGIGCI
       * :: *: ***********************   *******************.******

Pae    SRLALRDAFRRGSLVPVETPGLDLRRQFYFIWHKQKYQTATMREFLDLCRSETAGISRSD
Ppu    SRLALRDAFRRGSLVPVETPELDLLRQFYFIWHKQKYQTSAMREFLELCRNFTAGFTRSD
Avi    SRLALRDAFRRGSLVPVETPQLDLKRQFCFLWHKQKLHTAAMREFLEQCRALTAGVRRSD
       ******************** *** *** *:***** :*::*****: **  ***. ***

Pae    EIVLPLIP
Ppu    EIVLPPIA
Avi    EIVLPPIA
       ***** *.
```

S1 Fig. Multiple amino acid sequence alignment of *P. aeruginosa* FprA and FinR.
